# Association between Body Mass Index, Asymmetric Dimethylarginine and Risk of Cardiovascular Events and Mortality in Norwegian Patients with Suspected Stable Angina Pectoris

**DOI:** 10.1371/journal.pone.0152029

**Published:** 2016-03-22

**Authors:** Heidi Borgeraas, Jens Kristoffer Hertel, Gard Frodahl Tveitevåg Svingen, Eva Ringdal Pedersen, Reinhard Seifert, Ottar Nygård, Jøran Hjelmesæth

**Affiliations:** 1 Morbid Obesity Center, Vestfold Hospital Trust, Tønsberg, Norway; 2 Department of Endocrinology, Morbid Obesity and Preventive Medicine Institute of Clinical Medicine University of Oslo, Oslo, Norway; 3 Department of Clinical Science, University of Bergen, Bergen, Norway; 4 Department of Heart Disease, Haukeland University Hospital, Bergen, Norway; 5 KG Jebsen Center for Diabetes Research, Haukeland University Hospital, Bergen, 5021, Norway; University of Bologna, ITALY

## Abstract

**Background:**

Asymmetric dimethylarginine (ADMA) is associated with increased risk of atherosclerotic cardiovascular disease and mortality through inhibition of nitrogen oxide (NO) synthesis. As positive correlations between serum concentrations of NO and body mass index (BMI) have been observed, we aimed to explore whether the potential associations between plasma ADMA levels and the risk of acute myocardial infarction (AMI) and mortality were modified by BMI.

**Methods:**

Multivariable Cox proportional hazard models were used to estimate the hazard ratios (HR) for AMI, cardiovascular death and all-cause mortality according to baseline plasma ADMA levels in 4122 patients with suspected stable angina pectoris. Analyses were subsequently repeated in patients with BMI below (low BMI) or above (high BMI) median.

**Results:**

A total of 2982 patients (72%) were men. Median (range) age, plasma ADMA level and BMI were 62 (21–88) years, 0.54 (0.10–1.25) μmol/L and 26.3 (18.5–54.3) kg/m^2^, respectively. During a mean (standard deviation) follow-up time of 4.7 (1.4) years, 337 (8%) patients suffered from an AMI, 300 (7%) died, whereof 165 (55%) due to cardiovascular disease. Each 0.1 μmol/L increment in plasma ADMA level was associated with an increased risk of AMI (HR (95% CI) 1.21 (1.08, 1.35) and cardiovascular death 1.30 (1.13, 1.49) in participants with low BMI only. Interactions were significant for AMI (*p* = 0.04) and CV death (*p* = 0.03). BMI did not modify the association between plasma ADMA levels and all-cause mortality.

**Conclusion:**

Plasma ADMA levels were associated with risk of AMI and cardiovascular death among patients with low BMI only.

## Introduction

Plasma asymmetric dimethylarginine (ADMA) is recognized as a biomarker of atherosclerotic cardiovascular (CV) disease risk and mortality [1]. ADMA acts as a non-selective inhibitor of the nitrogen oxide synthases (NOS) and may increase the risk of CV complications through reduced synthesis of nitrogen oxide (NO).

NO, which is synthesized in small amounts by endothelial NOS (eNOS) during basal conditions, is an essential component for endothelial function and mediates endothelial vasodilatation, inhibits platelet aggregation and leukocyte adhesion to the endothelium and regulates myocardial contractility [2]. In acute and chronic inflammatory processes, NO levels might, however, become excessively high due to activation of inducible NOS (iNOS) [3]. In elevated quantities, NO reacts readily with other free radicals, increasing nitrosative and oxidative stress and potentially leading to injury of both the endothelium and myocytes [4–7].

Overweight and obesity are associated with chronic low grade inflammation [8]. The excess adipose tissue may cause accumulation of pro-inflammatory macrophages and subsequent induction of iNOS expression [9,10]. Notably, increased serum NO concentrations and markers of nitrosative and oxidative stress, have been observed in overweight and obese individuals, as compared to normal weight controls [11–14].

Due to the detrimental effects of high NO levels, an increased ADMA production might theoretically be protective in conditions associated with increased levels of iNOS-derived NO. Thus, in a large cohort of Norwegian patients with suspected stable angina pectoris we aimed to investigate whether the potential associations between plasma ADMA levels and risk of AMI, CV death and all-cause mortality were modified by BMI.

## Materials and Methods

### Study design, setting and population

A detailed description of the patients included in the present investigation has previously been published [15]. In short, two university hospitals in Western Norway recruited 4164 patients undergoing coronary angiography for suspected stable angina pectoris during the period from January 2000 to April 2004. Of these patients, 2573 (61.8%) were enrolled in the Western Norway B Vitamin Intervention Trial (WENBIT) (ClinicalTrials.gov Identifier: NCT00354081) which studied the effect of B-vitamin intervention on incident CV events and mortality [16].

Patients characterized as underweight (BMI<18.5) (n = 30) or those with missing data on BMI or ADMA (n = 11) were excluded. One patient with an extremely high plasma ADMA level, relative to the other patients was excluded as well, resulting in a total of 4122 subjects eligible for analysis. Data on plasma symmetric dimethylarginine (SDMA) were available from the WENBIT subpopulation (n = 2551).

The study protocol met the mandate of the Declaration of Helsinki and was approved by the Regional Committee for Medical and Health Research Ethics and the Norwegian Data Inspectorate. Written informed consent was obtained from all participants.

### Baseline variables

Each patient provided information about medical history, risk factors, and medications through a self-administered questionnaire, and all information was subsequently validated against medical records, as previously reported [16]. Height and weight were measured in light clothing at baseline by trained personnel, and BMI was calculated by dividing weight by height squared (kg/m^2^). Fasting referred to not having ingested food at least 6 hour prior to blood sample collection. Diabetes mellitus included type 1 and 2. Self-reported current smokers, those who quit smoking within <1 month prior to examination and patients with plasma cotinine >85 ng/mL were regarded as current smokers [17]. Left ventricular ejection fraction (LVEF) and extent of coronary artery disease (CAD) were assessed as previously described [18].

Blood samples were collected by study personnel prior to angiography and stored at -80°C until analysis. Patients were not requested to fast. Plasma ADMA and SDMA were determined by high performance liquid chromatography/tandem mass spectrometry (LC-MS/MS) at BEVITAL AS, Bergen, Norway (www.bevital.no), and the within-day coefficient of variation was 5–7% for ADMA and 8–9% for SDMA. Methods for measurement of serum apolipoprotein A-I (apoA-I), apolipoprotein B (apoB), lipoprotein (a) (Lp(a)), C-reactive protein (CRP), plasma cotinine, homocysteine [19] and calculation of LDL cholesterol and estimated glomerular filtration rate (eGFR) have previously been reported[18].

### End points and follow up

The patients were followed from angiography until either they experienced an AMI (fatal and non-fatal), died or throughout December 31^th^ 2006.

Clinical events information was collected as previously described [20]. An event was classified as fatal if death occurred within 28 days after onset. AMI was classified according to the diagnostic criteria of the revised AMI definition published in 2000 [21]. CV death included causes of death coded I00-I99 or R96, according to the International Statistical Evaluation of Disease, 10^th^ Revision System. All events were adjudicated by an endpoint committee who had no information on baseline biochemical characteristic.

### Statistical methods

Continuous variables are presented as means (± standard deviation (SD)) or medians (range) and categorical variables are reported as counts (percentage). Between-group differences were tested with independent samples t-test for continuous variables and chi-square test for categorical variables. Non-normally distributed variables (diastolic blood pressure, plasma homocysteine, serum creatinine, CRP, glucose, triglycerides and Lp(a)) were log transformed before analysis. Correlation analyses between ADMA and continuous and dichotomous variables were performed by calculating the Pearson product moment correlation coefficient and the point-biserial correlation coefficient, respectively. Adjusted correlations were carried out by calculating the partial correlation coefficient.

Cox proportional hazard models were used to calculate hazard ratios (HR) and 95% confidence intervals (95% CI) for incident AMI, CV death and all-cause mortality per 0.1 μmol/L increase in plasma ADMA levels. Proportionality assumptions were tested by visual examination of log minus log plots and by calculating Schoenfeld residuals. Selection of covariates in the multivariate adjusted model were based on clinical relevance the change in estimate method [22], and included age (years), sex, diabetes mellitus (yes/no), current smoking (yes/no), statin treatment (yes/no), homocysteine (μmol/L), hemoglobin (g/dL), apoB/apoA-I ratio and Lp(a) (mmol/L) (model 1). We considered systolic and diastolic blood pressure (mmHg), impaired LVEF (yes/no), the extent of significant CAD (0–3), eGFR (mL/min), use of beta blockers (yes/no), ACE-inhibitors (yes/no) and loop diuretics (yes/no) to be possible mediators of the effect of ADMA and did for that reason not include any of these variables in the main multivariate model (model 1) [23]. The variables were, however, added to an additional multivariate model (model 2). Vitamin B6 (yes/no) or folate/B12 (yes/no) WENBIT intervention status had no significant effect on the estimates (data not shown). Univariate, age and sex adjusted and multivariate adjusted HRs (95% CI) for incident AMI, CV death and all-cause mortality per 0.1 μmol/L increase in plasma SDMA levels were also calculated.

BMI was grouped according to the median value and patients with BMI equal to or below and above median were classified as “low BMI” and “high BMI”, respectively. The possible interaction between BMI and ADMA was examined by this stratification and by including the interaction product term of BMI (low and high BMI) and plasma ADMA (continuous) in the Cox model. The possible effect modification by BMI on the associations between plasma SDMA and incident AMI, CV death and all-cause mortality was also examined.

Non-linear effects were additionally investigated with generalized additive model (GAM) plots using penalized smoothing splines for the functional form of the covariate [24]. Potential breakpoints in the loglinear proportional hazards model were investigated with segmented regression (R-package segmented version 0.5–1.1).

All probability values are 2-tailed, and considered significant when <0.05. Statistical analyses were performed with SPSS 18 (SPSS Inc, Chicago, IL) and R 2.14.2 (The R-Foundation for Statistical Computing, Vienna, Austria).

## Results

### Baseline characteristics

For the 4122 patients included in the cohort, the mean (SD) age was 62 (10) years and 72% were males. The median (range) BMI was 26.3 (18.5–54.3) kg/m^2^ and plasma ADMA level was 0.54 (0.10–1.25) μmol/L.

Baseline characteristics of the study population are presented in Table 1. As compared with patients with high BMI (>26.3 kg/m^2^), the low BMI patient group (≤26.3 kg/m^2^) was on average 2 years older, less often fasting, included more frequently current smokers and had higher mean plasma ADMA and arginine levels. Those with low BMI had lower levels of atherogenic lipids, blood glucose, CRP, lower blood pressure and eGFR, and made up a lower proportion with diabetes. Patients with high BMI were more often treated with statins, beta blockers, ACE-inhibitors and loop diuretics at discharge from the hospital.

**Table 1:** Baseline characteristics in the total population and according to patients with low and high BMI.

	Total	Low BMI^a^	High BMI^b^	
	26.3 (18.5–54.3)^c^	24.2 (18.5–26.3)	29.0 (26.4–54.3)	
	n = 4122	n = 2061	n = 2061	p-value
**Demographic characteristics**				
Male sex, n (%)	2982 (72.3)	1471 (71.4)	1511 (73.3)	0.16
Age (years), mean (SD)	62 (10)	63 (10)	61 (10)	<0.001
Fasting, n(%)	1097(26.6)	511 (24.8)	586 (28.4)	<0.01
**Clinical characteristics**				
Systolic blood pressure (mmHg), mean (SD)	141 (21)	140 (21)	142 (21)	<0.01
Diastolic blood pressure (mmHg), mean (SD)	81 (10)	80 (10)	83 (10)	<0.001
Impaired Left ventricular ejection fraction, n (%)	534 (13.0)	265 (12.9)	269 (13.1)	0.86
**Cardiovascular risk factors, n (%)**				
Diabetes	494 (12.0)	175 (8.5)	319 (15.5)	<0.001
Current smoker	1056 (25.7)	584 (28.4)	472 (23.0)	<0.001
Ex smoker	1930 (46.9)	900 (43.7)	1030 (50.1)	<0.001
Never smoked	1129 (27.4)	576 (28.0)	553 (26.9)	0.45
**Cardiovascular history, n (%)**				
Previous acute myocardial infarction	1668 (40.5)	833 (40.4)	835 (40.5)	0.95
Previous cerebrovascular disease	285 (6.9)	137 (6.6)	148 (7.2)	0.50
Previous peripheral vascular disease	371 (9.0)	203 (9.8)	168 (8.2)	0.06
Previous percutaneous coronary intervention	793 (19.2)	375 (18.2)	418 (20.3)	0.10
Previous coronary artery bypass graft surgery	477 (11.6)	225 (10.9)	252 (12.2)	0.19
**Extent of coronary artery disease at baseline coronary angiography, n (%)**				
No significant coronary artery disease	1028 (24.9)	524 (25.4)	504 (24.5)	0.47
1 vessel disease	957 (23.2)	472 (22.9)	485 (23.5)	0.63
2 vessel disease	921 (22.3)	457 (22.1)	464 (22.5)	0.79
3 vesseldisease	1216 (29.5)	608 (29.5)	608 (29.5)	1.00
**Medication following baseline coronary angiography, n (%)**				
Acetylsalisylic acid	3369 (81.7)	1675 (81.3)	1693 (82.1)	0.47
Statins	3303 (80.1)	1610 (78.1)	1693 (82.1)	<0.01
Beta blockers	2988 (72.5)	1454 (70.5)	1534 (74.4)	<0.01
ACE-inhibitors	856 (20.8)	372 (18.0)	484 (23.5)	<0.001
Loop diuretics	447 (10.8)	188 (9.1)	259 (12.6)	<0.001
**Biochemical markers, mean (SD)**				
ADMA (μmol/L)	0.56 (0.12)	0.57 (0.12)	0.55 (0.11)	<0.01
Arginine (μmol/L)	78.9 (22.7)	80.0 (23.1)	77.8 (22.2)	<0.01
Homocysteine (μmol/L)	11.4 (4.94)	11.5 (4.87)	11.2 (5.01)	0.02
Creatinine (μmol/L)	92.6 (31.0)	92.9 (33.5)	92.3 (28.2)	0.54
eGFR (mL/min)	87.8 (17.2)	86.7 (17.4)	88.9 (17.0)	<0.001
CRP (mg/L)	3.69 (7.17)	3.38 (7.61)	4.01 (6.69)	<0.01
Glucose (mmol/L)	6.35 (2.40)	6.00 (2.27)	6.71 (2.49)	<0.001
HbA1c (mmol/L)	6.22 (1.39)	6.11 (1.32)	6.33 (1.44)	<0.001
Hemoglobin (g/dL)	14.3 (1.24)	14.1 (1.22)	14.4 (1.23)	<0.001
ApoA-I (g/L)	1.32 (0.27)	1.35 (0.28)	1.28 (0.25)	<0.001
ApoB (g/L)	0.90 (0.25)	0.88 (0.24)	0.93 (0.26)	<0.001
ApoB/ApoA ratio	0.71 (0.25)	0.68 (0.24)	0.75 (0.25)	<0.001
Total Cholesterol (mmol/L)	5.06 (1.17)	5.03 (1.11)	5.10 (1.22)	0.04
LDL cholesterol (mmol/L)	3.09 (1.03)	3.06 (1.00)	3.12 (1.05)	0.08
HDL cholesterol (mmol/L)	1.29 (0.37)	1.37 (0.40)	1.21 (0.33)	<0.001
Triglycerides (mmol/L)	1.78 (1.22)	1.53 (1.00)	2.03 (1.36)	<0.001
Lp(a) (mmol/L)	0.42 (0.39)	0.42 (0.39)	0.42 (0.38)	0.41

ACE, angiotensin-converting enzyme; ADMA, asymmetric dimethylarginine; apoA1, apolipoprotein A-I; apoB, apolipoprotein B; BMI, body mass index; CRP, c-reactive protein; eGFR, esitimated glomerular filtration rate; HDL, high density lipoprotein; LDL, low density lipoprotein; Lp(a), lipoprotein (a); SD, standard deviation

^a^ Equal to or below median (26.3 kg/m^2^) BMI

^b^ Above median (26.3 kg/m^2^) BMI

^c^ Median (range) BMI

Baseline plasma ADMA levels were correlated with female gender, older age, a history of peripheral vascular disease, use of loop diuretics, plasma homocysteine and serum creatinine levels, and inversely correlated with BMI, use of statins, eGFR, serum hemoglobin and Lp(a) (S1 Table). The correlation between plasma ADMA levels and BMI was no longer significant after adjustment for age and sex (data not shown).

Baseline plasma SDMA was measured in a subpopulation (n = 2551), and the mean (SD) plasma SDMA was 0.56 (0.15) μmol/L. Median BMI was 26.5kg/m^2^, and the mean (SD) plasma SDMA was significantly (p>0.001) higher among those with low BMI (0.58 (0.15) μmol/L) compared to those with high BMI (0.54 (0.14) μmol/L). Plasma SDMA levels were correlated with BMI (r = -0.15, p<0.01) and plasma ADMA levels (r = 0.33, p<0.01).

### End points and follow-up

During a mean (SD) follow-up of 4.7 (1.4) years, 337 (8%) patients experienced an AMI, of which 101 (30%) were fatal. A total of 300 (7%) patients died whereof 165 (55%) of these fatalities were due to CV disease.

### Associations between ADMA, AMI and mortality

Each 0.1 μmol/L increase in plasma ADMA level was associated with an increased risk of AMI (HR (95% CI) 1.12 (1.03, 1.22)), CV death (1.20 (1.07, 1.33)) and all-cause mortality; (1.21 (1.11, 1.31)) (Table 2, model 1). The associations were linear with no significant break-points (data not shown). After further adjustments for possible mediators of ADMA, the associations between plasma ADMA and risk of AMI (1.07 (0.98, 1.17)) and CV death (1.12 (0.99, 1.26)) were attenuated (Table 2, model 2).

**Table 2:** Risk of acute myocardial infarction, cardiovascular death and all-cause mortality according to plasma ADMA levels in the total population and in patients with high or low BMI.

Patients	Model						
		Acute myocardial infarction		Cardiovascular death		All-cause mortality	
		HR (95% CI)	p-value	HR (95% CI)	p-value	HR (95% CI)	p-value
**Total population**	**Univariate**						
	*ADMA (per 0*.*1* μmol*/L increase)*	1.20 (1.11, 1.30)	<0.001	1.36 (1.23, 1.50)	<0.001	1.35 (1.25, 1.45)	<0.001
	**Age and sex adjusted**						
	*ADMA (per 0*.*1* μmol*/L increase)*	1.15 (1.06, 1.24)	<0.01	1.25 (1.13, 1.39)	<0.001	1.26 (1.17, 1.36)	<0.001
	**Multivariate adjusted**						
	**Model 1**^a^						
	*ADMA (per 0*.*1* μmol*/L increase)*	1.12 (1.03, 1.22)	<0.01	1.20 (1.07, 1.33)	0.001	1.21 (1.11, 1.31)	<0.001
	**Model 2**^b^						
	*ADMA (per 0*.*1* μmol*/L increase)*	1.07 (0.98, 1.17)	0.15	1.12 (0.99, 1.26)	0.06	1.16 (1.07, 1.27)	<0.01
**Low BMI**^c^	**Univariate**						
	*ADMA (per 0*.*1* μmol/L increase)	1.29 (1.16, 1.42)	<0.001	1.46 (1.29, 1.65)	<0.001	1.39 (1.26, 1.53)	<0.001
	**Age and sex adjusted**						
	*ADMA (per 0*.*1* μmol/L increase)	1.23 (1.11, 1.37)	<0.001	1.36 (1.19, 1.54)	<0.001	1.31 (1.18, 1.45)	<0.001
	**Multivariate adjusted**						
	**Model 1**^a^						
	*ADMA (per 0*.*1* μmol*/L increase)*	1.21 (1.08, 1.35)	<0.01	1.30 (1.13, 1.49)	<0.001	1.26 (1.13, 1.40)	<0.001
	**Model 2**^b^						
	*ADMA (per 0*.*1* μmol*/L increase)*	1.12 (1.00, 1.27)	0.07	1.25 (1.07, 1.46)	<0.01	1.25 (1.11, 1.41)	<0.001
**High BMI**^d^	**Univariate**						
	*ADMA (per 0*.*1* μmol/L increase)	1.10 (0.97, 1.24)	0.13	1.22 (1.03, 1.44)	0.02	1.29 (1.15, 1.45)	<0.001
	**Age and sex adjusted**						
	*ADMA (per 0*.*1* μmol/L increase)	1.06 (0.93, 1.20)	0.40	1.13 (0.95, 1.34)	0.18	1.20 (1.02, 1.35)	<0.01
	**Multivariate adjusted**						
	**Model 1**^a^						
	*ADMA (per 0*.*1* μmol*/L increase)*	1.02 (0.90, 1.16)	0.72	1.06 (0.89, 1.27)	0.53	1.14 (1.00, 1.29)	0.04
	**Model 2**^b^						
	*ADMA (per 0*.*1* μmol*/L increase)*	1.00 (0.90, 1.14)	0.98	1.00 (0.82, 1.21)	0.96	1.09 (0.95, 1.24)	0.22

ADMA: asymmetric dimethylarginine; BMI: body mass index; CI: confidence interval; HR: hazard ratio;

^a^ Age (years), sex, diabetes mellitus (yes/no), current smoking (yes/no), statin treatment (yes/no), homocysteine (μmol/L), hemoglobin (g/dL), apoB/apoA-I ratio and Lp(a) (mmol/L)

^b^ Age (years), sex, diabetes mellitus (yes/no), current smoking (yes/no), statin treatment (yes/no), homocysteine (μmol/L), hemoglobin (g/dL), apoB/apoA-I ratio and Lp(a) (mmol/L) diastolic blood pressure (mmHg), systolic blood pressure (mmHg), treatment with beta blockers (yes/no), extent of significant CAD (0–3), estimated glomerular filtrationrate (mL/min), loop diuretics (yes/no), ACE-inhibitors (yes/no) and impaired left ventricular ejection fraction (yes/no)

^c^ Equal to or below median (26.3 kg/m^2^) BMI

^d^ Above median (26.3 kg/m^2^) BMI

One patient was excluded from the study based on extreme plasma levels of ADMA relative to the other patients (10 SD above mean). Inclusion of this outlier yielded similar results (data not shown).

Among those with data on plasma SDMA (n = 2551), the associations between plasma SDMA levels and risk of AMI, CV death and all-cause mortality were comparable to the associations between ADMA and endpoints (S2 Table).

### Stratification by BMI

**Fig 1:**
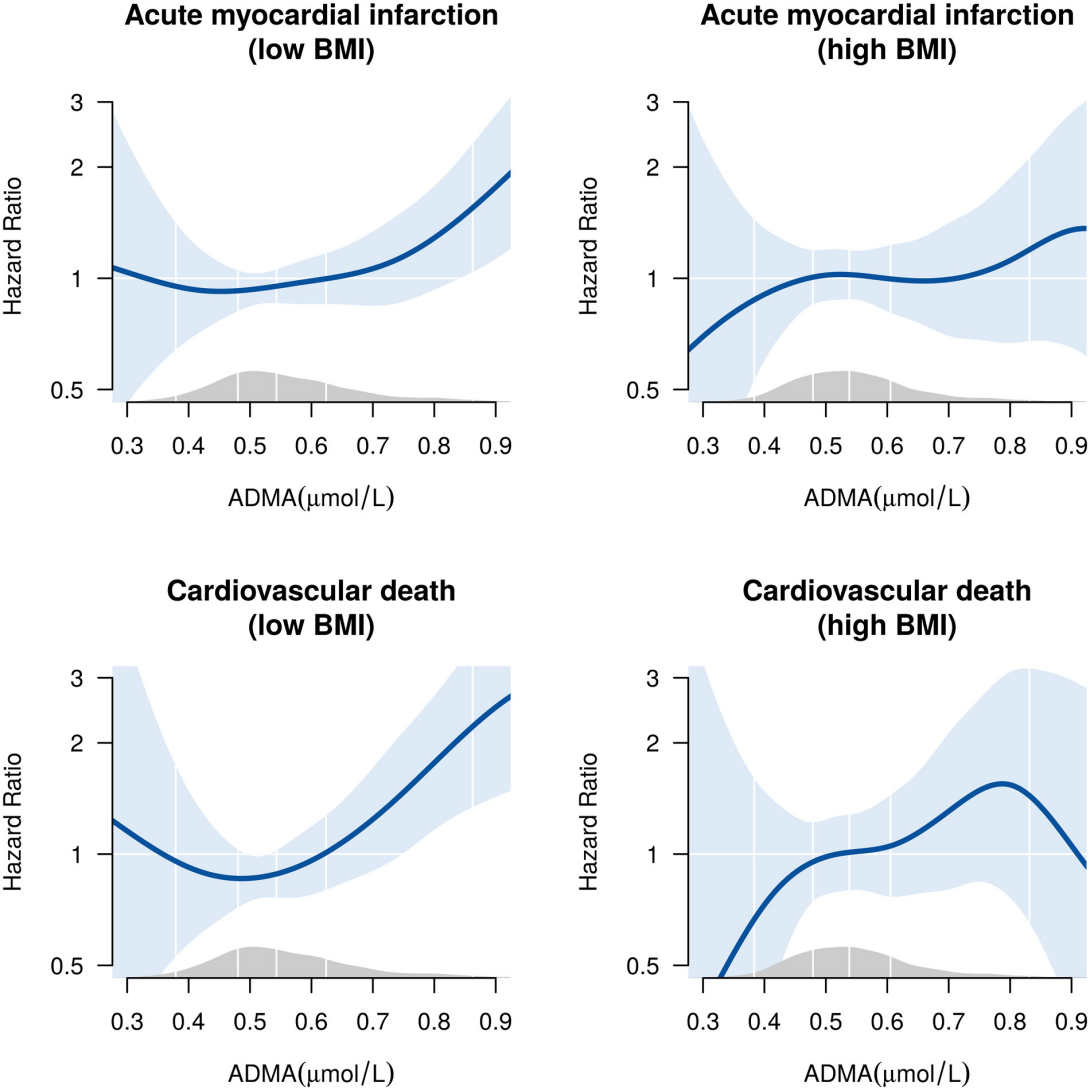
Plasma asymmetric dimethylarginine and risk of acute myocardial infarction and cardiovascular death in patients with low vs. high BMI. Association between plasma asymmetric dimethylarginine (ADMA) levels (μmol/L) and acute myocardial infarction (upper panels) and cardiovascular death (lower panels) in patients with body mass index (BMI) equal to or below median BMI (26.3 kg/m^2^) (low BMI) and BMI above median (high BMI). The nonlinear smoothing splines estimate of the hazard ratio were estimated with additive Cox proportional hazard regression models and adjusted for age (years), sex, diabetes mellitus (yes/no), current smoking (yes/no), statin treatment (yes/no), homocysteine (μmol/L), hemoglobin (g/dL) and apoB/apoA-I ratio and Lp(a) (mmol/L). The density plot along the X-axis shows the distribution of plasma ADMA.

BMI level modified the risk association between plasma ADMA and AMI (*p* for interaction = 0.04) and CV death (*p* for interaction = 0.03), but not all-cause mortality (*p* for interaction = 0.15). Each 0.1 μmol/L increase in plasma ADMA was associated with an increased risk of AMI and CV death in participants with low BMI only; HR (95% CI) 1.21 (1.08, 1.35) and 1.30 (1.13, 1.49), respectively (Table 2 and Fig 1). After further adjustments (model 2), the interactions between plasma ADMA and BMI with regards to risk of AMI (*p* for interaction = 0.05) and CV death (*p* for interaction = 0.031) remained significant, while the interaction between plasma ADMA and BMI with regards to all-cause mortality was still non-significant (*p* for interaction = 0.09). Adjustment for CRP and fasting did not significantly influence the results (data not shown). Segmented Cox regression analyses revealed a linear association between plasma ADMA levels and endpoints, with no significant break-points (data not shown).

BMI did, however, not significantly modify the association between plasma SDMA levels and risk of AMI, CV death and all-cause mortality (S2 Table).

## Discussion

### Principal findings

In this large prospective cohort study of patients with suspected stable angina pectoris, increasing baseline plasma levels of ADMA were associated with higher risk of incident AMI, CV death and all-cause mortality. The associations between plasma ADMA levels and incident AMI and CV death were, however, significant in patients with low BMI (equal to or below median BMI (26.3 kg/m^2^)) only. The association between plasma ADMA level and all-cause mortality was not modified by BMI.

### ADMA and risk prediction

High plasma ADMA levels have been associated with increased risk of incident CV events and all-cause mortality in patients with stable coronary heart disease [25–28], following PCI [26], chronic heart failure [29], and recent AMI [30], peripheral arterial disease [31], hemodialysis patients [32], renal transplant recipients with multi organ-failure [33] diabetes mellitus[34,35] as well as in the general population [36–38].

To the best of our knowledge, this is, however, the first study to investigate the possible moderating effect of BMI on the association between plasma ADMA levels and the risk of AMI and mortality in a population of patients with suspected coronary artery disease.

### Possible explanations

The NO molecule can be thought of as a double edged sword; under physiological conditions NO is produced in low concentrations and participates in the maintenance of homeostasis in the cardiovascular, immune and central nervous systems [39]. However, during pathophysiologic conditions such as inflammation, iNOS may generate up to 1000 times the normal amount of NO [40] with subsequent production of reactive nitrogen and oxygen species [3]. Overweight and obesity are related to chronic low grade inflammation[8], elevated levels of NO and markers of nitrosative and oxidative stress [11–14]. As ADMA is a non-selective inhibitor of NOS, the lack of association observed between plasma ADMA levels and risk of AMI and CV death in overweight and obese patients in the current study can be put down to a possible beneficial regulatory effect of ADMA on the inflammatory processes and the elevated NO levels. We would thus expect the interaction between ADMA and BMI to be attenuated when adjusting for an inflammatory marker, but including CRP in the regression analyses did not influence this interaction. A possible explanation might be the extensive use of statins which are known to decrease CRP levels [41] and also increase NO levels [42].

In a subpopulation, increasing levels of SDMA were also associated with increased risk of AMI, CV death and all-cause mortality. In contrast to ADMA, SDMA does not directly inhibit NO synthesis, thus a beneficial regulatory effect on elevated NO levels by SDMA in overweight and obese individuals is unlikely. As expected, the associations between SDMA and endpoints were not modified by BMI.

On a similar note, a Framingham offspring study revealed that the predictive value of plasma ADMA levels for all-cause mortality was only evident in patients without diabetes, while the association appeared to disappear in those with diabetes [38]. BMI and diabetes are closely interrelated, and there were admittedly more diabetic patients in the high BMI group in the present study, yet there were no significant interactions between plasma ADMA levels and diabetes with regards to any of the end points. Furthermore, in the general male population, plasma ADMA levels have been found to be associated with increased risk of coronary events in non-smokers, but not in smokers [36]. No such interaction was observed in the current study. However, it is also worth considering that both hyperglycemia and cigarette smoke increases the formation of ROS and induces inflammation [43,44]; hence these effect modifications might potentially be explained by a similar mechanism to that suggested in this study.

Stratified analyses also revealed stronger risk associations of ADMA to all cause mortality among subjects with low BMI compared to those with high BMI. However, the interaction was not statistically relevant in relation to this endpoint.

### Strengths and limitations of the study

The study included a large and well-characterised population and had a prospective design. The effect of ADMA is thought to be mediated through reduced NO availability. Elevated ADMA levels may in turn result in increased blood pressure, cardiac dysfunction [45], increased progression of atherosclerosis [46,47] and reduced renal plasma flow and GFR [48–50]. The inclusion of variables reflecting any of these parameters might thus cause an underestimation of the effect of ADMA. We did, however, include possible mediators in an additional multivariate model, and as expected, the effect of ADMA on the risk of AMI and CV death was somewhat attenuated. The interaction between ADMA and BMI was, however, still significant.

A possible limitation to our study is the single baseline measurements of ADMA, which may mean that the true risk estimates were underestimated due to regression dilution bias [51]. Also, we cannot preclude the possibility of residual confounding, an inherent limitation in all observational research.

### Conclusion

We confirmed previous findings of a positive association between plasma ADMA levels and incident AMI, CV death and all-cause mortality. The associations between plasma ADMA levels and risk of AMI and CV death were, however, confined to patients with low BMI only. In contrast, BMI had no modifying effect on the relationship between plasma AMA levels and all-cause mortality. More research is warranted in order to corroborate our findings in other populations and to clarify the possible underlying mechanisms.

## Supporting Information

S1 Table Baseline characteristics and correlations with plasma ADMA levels. (DOCX)

S2 Table Risk of acute myocardial infarction, cardiovascular death and all-cause mortality according to plasma SDMA levels in the total population and in patients with high or low BMI. (DOCX)

## References

[1] Sibal L, Agarwal SC, Home PD, Boger RH (2010) The Role of Asymmetric Dimethylarginine (ADMA) in Endothelial Dysfunction and Cardiovascular Disease. Curr Cardiol Rev 6: 82–90. 10.2174/157340310791162659 21532773 PMC2892080

[2] Loscalzo J, Welch G (1995) Nitric oxide and its role in the cardiovascular system. Prog Cardiovasc Dis 38: 87–104. 7568906 10.1016/s0033-0620(05)80001-5

[3] Bogdan C (2001) Nitric oxide and the immune response. Nat Immunol 2: 907–916. 11577346 10.1038/ni1001-907

[4] Muhl H, Sandau K, Brune B, Briner VA, Pfeilschifter J (1996) Nitric oxide donors induce apoptosis in glomerular mesangial cells, epithelial cells and endothelial cells. Eur J Pharmacol 317: 137–149. 8982730 10.1016/s0014-2999(96)00701-7

[5] Pinsky DJ, Cai B, Yang X, Rodriguez C, Sciacca RR, Cannon PJ (1995) The lethal effects of cytokine-induced nitric oxide on cardiac myocytes are blocked by nitric oxide synthase antagonism or transforming growth factor beta. J Clin Invest 95: 677–685. 7532189 10.1172/JCI117713 PMC295534

[6] Ing DJ, Zang J, Dzau VJ, Webster KA, Bishopric NH (1999) Modulation of cytokine-induced cardiac myocyte apoptosis by nitric oxide, Bak, and Bcl-x. Circ Res 84: 21–33. 9915771 10.1161/01.res.84.1.21

[7] Turrens JF (2003) Mitochondrial formation of reactive oxygen species. J Physiol 552: 335–344. 14561818 10.1113/jphysiol.2003.049478 PMC2343396

[8] Gregor MF, Hotamisligil GS (2011) Inflammatory mechanisms in obesity. Annu Rev Immunol 29: 415–445. 10.1146/annurev-immunol-031210-101322 21219177

[9] Trayhurn P, Wood IS (2005) Signalling role of adipose tissue: adipokines and inflammation in obesity. Biochem Soc Trans 33: 1078–1081. 16246049 10.1042/BST0331078

[10] Tzanavari T, Giannogonas P, Karalis KP (2010) TNF-alpha and obesity. Curr Dir Autoimmun 11: 145–156. 10.1159/000289203 20173393

[11] Choi JW, Pai SH, Kim SK, Ito M, Park CS, Cha YN (2001) Increases in nitric oxide concentrations correlate strongly with body fat in obese humans. Clin Chem 47: 1106–1109. 11375300

[12] Olszanecka-Glinianowicz M, Zahorska-Markiewicz B, Janowska J, Zurakowski A (2004) Serum concentrations of nitric oxide, tumor necrosis factor (TNF)-alpha and TNF soluble receptors in women with overweight and obesity. Metabolism 53: 1268–1273. 15375781 10.1016/j.metabol.2004.07.001

[13] Codoner-Franch P, Tavarez-Alonso S, Murria-Estal R, Megias-Vericat J, Tortajada-Girbes M, Alonso-Iglesias E (2011) Nitric oxide production is increased in severely obese children and related to markers of oxidative stress and inflammation. Atherosclerosis 215: 475–480. 10.1016/j.atherosclerosis.2010.12.035 21300354

[14] Paik JK, Kim M, Yen Y, Ahn HY, Lee SH, Lee JH (2015) Circulating Lp-PLA(2) activity correlates with oxidative stress and cytokines in overweight/obese postmenopausal women not using hormone replacement therapy. Age (Dordr) 37: 32. 25840804 10.1007/s11357-015-9770-4 PMC4385327

[15] Svingen GF, Ueland PM, Pedersen EK, Schartum-Hansen H, Seifert R, Ebbing M, et al (2013) Plasma Dimethylglycine and Risk of Incident Acute Myocardial Infarction in Patients With Stable Angina Pectoris. Arterioscler Thromb Vasc Biol. 10.1161/ATVBAHA.113.301714 23723367

[16] Ebbing M, Bleie O, Ueland PM, Nordrehaug JE, Nilsen DW, Vollset SE, et al (2008) Mortality and cardiovascular events in patients treated with homocysteine-lowering B vitamins after coronary angiography: a randomized controlled trial. JAMA 300: 795–804. 10.1001/jama.300.7.795 18714059

[17] (2002) Biochemical verification of tobacco use and cessation. Nicotine Tob Res 4: 149–159. 12028847 10.1080/14622200210123581

[18] Borgeraas H, Hertel JK, Svingen GF, Seifert R, Pedersen EK, Schartum-Hansen H, et al (2014) Association of body mass index with risk of acute myocardial infarction and mortality in Norwegian male and female patients with suspected stable angina pectoris: a prospective cohort study. BMC Cardiovasc Disord 14: 68 10.1186/1471-2261-14-68 24885137 PMC4032453

[19] Bleie O, Refsum H, Ueland PM, Vollset SE, Guttormsen AB, Nexo E, et al (2004) Changes in basal and postmethionine load concentrations of total homocysteine and cystathionine after B vitamin intervention. Am J Clin Nutr 80: 641–648. 15321804 10.1093/ajcn/80.3.641

[20] Pedersen ER, Ueland T, Seifert R, Aukrust P, Schartum-Hansen H, Ebbing M, et al (2010) Serum osteoprotegerin levels and long-term prognosis in patients with stable angina pectoris. Atherosclerosis 212: 644–649. 10.1016/j.atherosclerosis.2010.06.027 20621297

[21] (2000) Myocardial infarction redefined—a consensus document of The Joint European Society of Cardiology/American College of Cardiology Committee for the redefinition of myocardial infarction. Eur Heart J 21: 1502–1513. 10973764 10.1053/euhj.2000.2305

[22] Greenland S (1989) Modeling and variable selection in epidemiologic analysis. Am J Public Health 79: 340–349. 2916724 10.2105/ajph.79.3.340 PMC1349563

[23] Schisterman EF, Cole SR, Platt RW (2009) Overadjustment bias and unnecessary adjustment in epidemiologic studies. Epidemiology 20: 488–495. 10.1097/EDE.0b013e3181a819a1 19525685 PMC2744485

[24] Therneau TM G P (2000) Modeling Survival Data—Extending the Cox Model. New York: Springer-Verlag.

[25] Borgeraas H, Strand E, Ringdal Pedersen E, Dierkes J, Ueland PM, Seifert R, et al (2012) Omega-3 Status and the Relationship between Plasma Asymmetric Dimethylarginine and Risk of Myocardial Infarction in Patients with Suspected Coronary Artery Disease. Cardiol Res Pract 2012: 201742 10.1155/2012/201742 23346455 PMC3549394

[26] Lu TM, Ding YA, Lin SJ, Lee WS, Tai HC (2003) Plasma levels of asymmetrical dimethylarginine and adverse cardiovascular events after percutaneous coronary intervention. Eur Heart J 24: 1912–1919. 14585249 10.1016/j.ehj.2003.08.013

[27] Schnabel R, Blankenberg S, Lubos E, Lackner KJ, Rupprecht HJ, Espinola-Klein C, et al (2005) Asymmetric dimethylarginine and the risk of cardiovascular events and death in patients with coronary artery disease: results from the AtheroGene Study. Circ Res 97: e53–59. 16100045 10.1161/01.RES.0000181286.44222.61

[28] Meinitzer A, Seelhorst U, Wellnitz B, Halwachs-Baumann G, Boehm BO, Winkelmann BR, et al (2007) Asymmetrical dimethylarginine independently predicts total and cardiovascular mortality in individuals with angiographic coronary artery disease (the Ludwigshafen Risk and Cardiovascular Health study). Clin Chem 53: 273–283. 17185364 10.1373/clinchem.2006.076711

[29] Duckelmann C, Mittermayer F, Haider DG, Altenberger J, Eichinger J, Wolzt M (2007) Asymmetric dimethylarginine enhances cardiovascular risk prediction in patients with chronic heart failure. Arterioscler Thromb Vasc Biol 27: 2037–2042. 17569878 10.1161/ATVBAHA.107.147595

[30] Zeller M, Korandji C, Guilland JC, Sicard P, Vergely C, Lorgis L, et al (2008) Impact of asymmetric dimethylarginine on mortality after acute myocardial infarction. Arterioscler Thromb Vasc Biol 28: 954–960. 10.1161/ATVBAHA.108.162768 18276906

[31] Mittermayer F, Krzyzanowska K, Exner M, Mlekusch W, Amighi J, Sabeti S, et al (2006) Asymmetric dimethylarginine predicts major adverse cardiovascular events in patients with advanced peripheral artery disease. Arterioscler Thromb Vasc Biol 26: 2536–2540. 16931791 10.1161/01.ATV.0000242801.38419.48

[32] Zoccali C, Bode-Boger S, Mallamaci F, Benedetto F, Tripepi G, Malatino L, et al (2001) Plasma concentration of asymmetrical dimethylarginine and mortality in patients with end-stage renal disease: a prospective study. Lancet 358: 2113–2117. 11784625 10.1016/s0140-6736(01)07217-8

[33] Nijveldt RJ, Teerlink T, Van Der Hoven B, Siroen MP, Kuik DJ, Rauwerda JA, et al (2003) Asymmetrical dimethylarginine (ADMA) in critically ill patients: high plasma ADMA concentration is an independent risk factor of ICU mortality. Clin Nutr 22: 23–30. 12553946 10.1054/clnu.2002.0613

[34] Lajer M, Tarnow L, Jorsal A, Teerlink T, Parving HH, Rossing P (2008) Plasma concentration of asymmetric dimethylarginine (ADMA) predicts cardiovascular morbidity and mortality in type 1 diabetic patients with diabetic nephropathy. Diabetes Care 31: 747–752. 18162497 10.2337/dc07-1762

[35] Krzyzanowska K, Mittermayer F, Wolzt M, Schernthaner G (2007) Asymmetric dimethylarginine predicts cardiovascular events in patients with type 2 diabetes. Diabetes Care 30: 1834–1839. 17456842 10.2337/dc07-0019

[36] Maas R, Schulze F, Baumert J, Lowel H, Hamraz K, Schwedhelm E, et al (2007) Asymmetric dimethylarginine, smoking, and risk of coronary heart disease in apparently healthy men: prospective analysis from the population-based Monitoring of Trends and Determinants in Cardiovascular Disease/Kooperative Gesundheitsforschung in der Region Augsburg study and experimental data. Clin Chem 53: 693–701. 17317881 10.1373/clinchem.2006.081893

[37] Leong T, Zylberstein D, Graham I, Lissner L, Ward D, Fogarty J, et al (2008) Asymmetric dimethylarginine independently predicts fatal and nonfatal myocardial infarction and stroke in women: 24-year follow-up of the population study of women in Gothenburg. Arterioscler Thromb Vasc Biol 28: 961–967. 10.1161/ATVBAHA.107.156596 18292394

[38] Boger RH, Sullivan LM, Schwedhelm E, Wang TJ, Maas R, Benjamin EJ, et al (2009) Plasma asymmetric dimethylarginine and incidence of cardiovascular disease and death in the community. Circulation 119: 1592–1600. 10.1161/CIRCULATIONAHA.108.838268 19289633 PMC2742491

[39] Bredt DS (1999) Endogenous nitric oxide synthesis: biological functions and pathophysiology. Free Radic Res 31: 577–596. 10630682 10.1080/10715769900301161

[40] Nathan C (1997) Inducible nitric oxide synthase: what difference does it make? J Clin Invest 100: 2417–2423. 9366554 10.1172/JCI119782 PMC508440

[41] Albert MA, Danielson E, Rifai N, Ridker PM, Investigators P (2001) Effect of statin therapy on C-reactive protein levels: the pravastatin inflammation/CRP evaluation (PRINCE): a randomized trial and cohort study. JAMA 286: 64–70. 11434828 10.1001/jama.286.1.64

[42] Kotamraju S, Williams CL, Kalyanaraman B (2007) Statin-induced breast cancer cell death: role of inducible nitric oxide and arginase-dependent pathways. Cancer Res 67: 7386–7394. 17671209 10.1158/0008-5472.CAN-07-0993

[43] Lee J, Taneja V, Vassallo R (2012) Cigarette smoking and inflammation: cellular and molecular mechanisms. J Dent Res 91: 142–149. 10.1177/0022034511421200 21876032 PMC3261116

[44] Giacco F, Brownlee M (2010) Oxidative stress and diabetic complications. Circ Res 107: 1058–1070. 10.1161/CIRCRESAHA.110.223545 21030723 PMC2996922

[45] Achan V, Broadhead M, Malaki M, Whitley G, Leiper J, MacAllister R, et al (2003) Asymmetric dimethylarginine causes hypertension and cardiac dysfunction in humans and is actively metabolized by dimethylarginine dimethylaminohydrolase. Arterioscler Thromb Vasc Biol 23: 1455–1459. 12805079 10.1161/01.ATV.0000081742.92006.59

[46] Loland KH, Bleie O, Borgeraas H, Strand E, Ueland PM, Svardal A, et al (2013) The association between progression of atherosclerosis and the methylated amino acids asymmetric dimethylarginine and trimethyllysine. PLoS One 8: e64774 10.1371/journal.pone.0064774 23734218 PMC3666971

[47] Heitzer T, Baldus S, von Kodolitsch Y, Rudolph V, Meinertz T (2005) Systemic endothelial dysfunction as an early predictor of adverse outcome in heart failure. Arterioscler Thromb Vasc Biol 25: 1174–1179. 15831810 10.1161/01.ATV.0000166516.52477.81

[48] Knowles JW, Reddick RL, Jennette JC, Shesely EG, Smithies O, Maeda N (2000) Enhanced atherosclerosis and kidney dysfunction in eNOS(-/-)Apoe(-/-) mice are ameliorated by enalapril treatment. J Clin Invest 105: 451–458. 10683374 10.1172/JCI8376 PMC289160

[49] Qiu C, Muchant D, Beierwaltes WH, Racusen L, Baylis C (1998) Evolution of chronic nitric oxide inhibition hypertension: relationship to renal function. Hypertension 31: 21–26. 9449385 10.1161/01.hyp.31.1.21

[50] Fujii H, Takiuchi S, Kawano Y, Fukagawa M (2008) Putative role of asymmetric dimethylarginine in microvascular disease of kidney and heart in hypertensive patients. Am J Hypertens 21: 650–656. 10.1038/ajh.2008.29 18443575

[51] Clarke R, Shipley M, Lewington S, Youngman L, Collins R, Marmot M, et al (1999) Underestimation of risk associations due to regression dilution in long-term follow-up of prospective studies. Am J Epidemiol 150: 341–353. 10453810 10.1093/oxfordjournals.aje.a010013

