# Supplementary material for: Association between Body Mass Index, Asymmetric Dimethylarginine and Risk of Cardiovascular Events and Mortality in Norwegian Patients with Suspected Stable Angina Pectoris
Source: PLoS One. 2016 Mar 22;11(3):e0152029. doi: 10.1371/journal.pone.0152029 (PMC4803210; doi:10.1371/journal.pone.0152029)
Supplement: S1 Table — (DOCX) [file pone.0152029.s001.docx]

| **Supporting Table 1: Baseline characteristics and correlations with plasma ADMA levels** | |
| --- | --- |
|  | **Correlation coefficientᵃ** |
|  |  |
| **Demographic characteristics** |  |
| Male sex | -0.08** |
| Age (years) | 0.20** |
| Fasting | -0.12** |
|  |  |
| **Clinical characteristics** |  |
| BMI (kg/m²) | -0.06** |
| Systolic blood pressure (mmHg) | -0.04* |
| Diastolic blood pressure (mmHg) | -0.05** |
| Impaired Left ventricular ejection fractionᵇ | 0.04* |
|  |  |
| **Cardiovascular risk factors** |  |
| Diabetesᵈ | -0.03 |
| Current smokerᵉ | -0.001 |
| Ex smoker | -0.02 |
| Never smoked | 0.02 |
|  |  |
| **Cardiovascular history** |  |
| Previous acute myocardial infarction | 0.03 |
| Previous cerebrovascular disease | 0.05** |
| Previous peripheral vascular disease | 0.11** |
| Previous percutaneous coronary intervention | -0.03* |
| Previous coronary artery bypass graft surgery | -0.02 |
|  |  |
| **Extent of coronary artery disease at baseline coronary angiography** | |
| No significant coronary artery disease | 0.05** |
| 1 vessel disease | -0.07** |
| 2 vessel disease | -0.03 |
| 3 vesseldisease | 0.03* |
|  |  |
| **Medication following baseline coronary angiography** | |
| Acetylsalisylic acid | -0.05** |
| Statins | -0.09** |
| Beta blockers | -0.01 |
| ACE-inhibitors | 0.05** |
| Loop diuretics | 0.13** |
|  |  |
| **Biochemical markers** |  |
| Arginine (µmol/L) | 0.06** |
| Homocysteine (µmol/L) | 0.22** |
| Creatinine (µmol/L) | 0.19** |
| eGFR (mL/min) | -0.32** |
| CRP (mg/L) | 0.06** |
| Glucose (mmol/L) | 0.02 |
| HbA1c (mmol/L) | 0.03 |
| Hemoglobin (g/dL) | -0.18** |
| ApoA-I (g/L) | 0.04** |
| ApoB (g/L) | 0.02 |
| ApoB/ApoA-I ratio | -0.01 |
| Total Cholesterol (mmol/L) | -0.01 |
| LDL cholesterol (mmol/L) | 0.02 |
| HDL cholesterol (mmol/L) | 0.02 |
| Triglycerides (mmol/L) | -0.06** |
| Lp(a) (mmol/L) | -0.08** |
| ADMA: asymmetric dimethylarginine; ACE: angiotensin-converting enzyme; ApoA: apolipoprotein A-I; ApoB: apolipoprotein B; BMI: body mass index; CRP: c-reactive protein; eGFR: esitimated glomerular filtration rate; HDL: high density lipoprotein; LDL: low density lipoprotein; Lp(a): lipoprotein (a)  a Pearson or point-biserial correlation coefficient | |
| * Correlation is significant at the 0.05 level (2-tailed) | |
| ** Correlation is significant at the 0.01 level (2-tailed) | |
