# Supplementary material for: Association between Body Mass Index, Asymmetric Dimethylarginine and Risk of Cardiovascular Events and Mortality in Norwegian Patients with Suspected Stable Angina Pectoris
Source: PLoS One. 2016 Mar 22;11(3):e0152029. doi: 10.1371/journal.pone.0152029 (PMC4803210; doi:10.1371/journal.pone.0152029)
Supplement: S2 Table — (DOCX) [file pone.0152029.s002.docx]

| **Supporting Table 2: Risk of acute myocardial infarction, cardiovascular death and all-cause mortality according to plasma SDMA levels in the total population and in patients with high or low BMI** | | | | | | | | |  |  |
| --- | --- | --- | --- | --- | --- | --- | --- | --- | --- | --- |
|  |  |  |  |  |  |  |  | | |  |
| **Patients** | **Model** |  | | | | | | | | |
|  |  | **Acute myocardial infarction** | | **Cardiovascular death** | | **All-cause mortality** | | | | |
|  |  | HR (95% CI) | p-value | HR (95% CI) | p-value | HR (95% CI) | | p-value | | |
|  |  |  |  |  |  |  | |  | | |
| **Total population** | **Univariate** |  |  |  |  |  | |  | | |
|  | *SDMA (per 0.1* µmol*/L increase)* | 1.21 (1.13, 1.30) | <0.001 | 1.38 (1.28, 1.48) | <0.001 | 1.31 (1.23, 1.40) | | <0.001 | | |
|  |  |  |  |  |  |  | |  | | |
|  | **Age and sex adjusted** |  |  |  |  |  | |  | | |
|  | *SDMA (per 0.1* µmol*/L increase)* | 1.18 (1.09, 1.27) | <0.001 | 1.27 (1.16, 1.40) | <0.001 | 1.20 (1.11, 1.30) | | <0.001 | | |
|  |  |  |  |  |  |  | |  | | |
|  | **Multivariate adjusted** |  |  |  |  |  | |  | | |
|  |  |  |  |  |  |  | |  | | |
|  | **Model 1^a^** |  |  |  |  |  | |  | | |
|  | *SDMA (per 0.1* µmol*/L increase)* | 1.14 (1.05, 1.24) | <0.01 | 1.25 (1.12, 1.40) | <0.001 | 1.15 (1.06, 1.26) | | <0.01 | | |
|  |  |  |  |  |  |  | |  | | |
|  | **Model 2^b^** |  |  |  |  |  | |  | | |
|  | *SDMA (per 0.1* µmol*/L increase)* | 1.05 (0.94, 1.17) | 0.38 | 1.10 (0.95, 1.28) | 0.19 | 1.10 (0.98, 1.24) | | 0.12 | | |
|  |  |  |  |  |  |  | |  | | |
| **Low BMI^c^** | **Univariate** |  |  |  |  |  | |  | | |
|  | *SDMA (per 0.1* µmol/L increase) | 1.25 (1.14, 1.38) | <0.001 | 1.41 (1.26, 1.58) | <0.001 | 1.32 (1.20, 1.45) | | <0.001 | | |
|  |  |  |  |  |  |  | |  | | |
|  | **Age and sex adjusted** |  |  |  |  |  | |  | | |
|  | *SDMA (per 0.1* µmol/L increase) | 1.23 (1.10, 1.37) | <0.001 | 1.26 (1.10, 1.45) | <0.01 | 1.18 (1.06, 1.32) | | <0.01 | | |
|  |  |  |  |  |  |  | |  | | |
|  | **Multivariate adjusted** |  |  |  |  |  | |  | | |
|  |  |  |  |  |  |  | |  | | |
|  | **Model 1^a^** |  |  |  |  |  | |  | | |
|  | *SDMA (per 0.1* µmol*/L increase)* | 1.23 (1.09, 1.39) | <0.01 | 1.29 (1.09, 1.53) | <0.01 | 1.13 (1.00, 1.28) | | 0.05 | | |
|  |  |  |  |  |  |  | |  | | |
|  | **Model 2^b^** |  |  |  |  |  | |  | | |
|  | *SDMA (per 0.1* µmol*/L increase)* | 1.05 (0.89, 1.22) | 0.59 | 0.98 (0.78, 1.22) | 0.83 | 1.06 (0.89, 1.26) | | 0.51 | | |
|  |  |  |  |  |  |  | |  | | |
| **High BMI^d^** | **Univariate** |  |  |  |  |  | |  | | |
|  | *SDMA (per 0.1* µmol/L increase) | 1.19 (1.08, 1.31) | <0.01 | 1.35 (1.22, 1.50) | <0.001 | 1.30 (1.18, 1.42) | | <0.001 | | |
|  |  |  |  |  |  |  | |  | | |
|  | **Age and sex adjusted** |  |  |  |  |  | |  | | |
|  | *SDMA (per 0.1* µmol/L increase) | 1.14 (1.02, 1.28) | 0.02 | 1.33 (1.15, 1.53) | <0.001 | 1.24 (1.10, 1.40) | | <0.001 | | |
|  |  |  |  |  |  |  | |  | | |
|  | **Multivariate adjusted** |  |  |  |  |  | |  | | |
|  |  |  |  |  |  |  | |  | | |
|  | **Model 1^a^** |  |  |  |  |  | |  | | |
|  | *SDMA (per 0.1* µmol*/L increase)* | 1.09 (0.97, 1.22) | 0.13 | 1.25 (1.08, 1.45) | <0.01 | 1.19 (1.05, 1.36) | | 0.01 | | |
|  |  |  |  |  |  |  | |  | | |
|  | **Model 2^b^** |  |  |  |  |  | |  | | |
|  | *SDMA (per 0.1* µmol*/L increase)* | 1.08 (0.92, 1.25) | 0.33 | 1.27 (1.02, 1.59) | 0.03 | 1.19 (1.00, 1.42) | | 0.07 | | |
| SDMA: symmetric dimethylarginine; BMI: body mass index; CI: confidence interval; HR: hazard ratio; | | | | | | | | | | |
| a Age (years), sex, diabetes mellitus (yes/no), current smoking (yes/no), statin treatment (yes/no), homocysteine (µmol/L), hemoglobin (g/dL), apoB/apoA-I ratio and Lp(a) (mmol/L) | | | | | | | | | | |
| b Age (years), sex, diabetes mellitus (yes/no), current smoking (yes/no), statin treatment (yes/no), homocysteine (µmol/L), hemoglobin (g/dL), apoB/apoA-I ratio and Lp(a) (mmol/L) diastolic blood pressure (mmHg), systolic blood pressure (mmHg), treatment with beta blockers (yes/no), extent of significant CAD (0-3), estimated glomerular filtrationrate (mL/min), loop diuretics (yes/no), ACE-inhibitors (yes/no) and impaired left ventricular ejection fraction (yes/no)  c Equal to or below median (26.5 kg/m^2^) BMI  d Above median (26.5 kg/m^2^) BMI | | | | | | | | | | |
